# A COVID-19 medical image classification algorithm based on Transformer

**DOI:** 10.1038/s41598-023-32462-2

**Published:** 2023-04-01

**Authors:** Keying Ren, Geng Hong, Xiaoyan Chen, Zichen Wang

**Affiliations:** College of Electronic Information and Automation, Tianjin University of Science and Technology, Tianjin, 300222 China

**Keywords:** Computational biology and bioinformatics, Health care, Health occupations, Engineering

## Abstract

Coronavirus 2019 (COVID-19) is a new acute respiratory disease that has spread rapidly throughout the world. This paper proposes a novel deep learning network based on ResNet-50 merged transformer named RMT-Net. On the backbone of ResNet-50, it uses Transformer to capture long-distance feature information, adopts convolutional neural networks and depth-wise convolution to obtain local features, reduce the computational cost and acceleration the detection process. The RMT-Net includes four stage blocks to realize the feature extraction of different receptive fields. In the first three stages, the global self-attention method is adopted to capture the important feature information and construct the relationship between tokens. In the fourth stage, the residual blocks are used to extract the details of feature. Finally, a global average pooling layer and a fully connected layer perform classification tasks. Training, verification and testing are carried out on self-built datasets. The RMT-Net model is compared with ResNet-50, VGGNet-16, i-CapsNet and MGMADS-3. The experimental results show that the RMT-Net model has a Test_ acc of 97.65% on the X-ray image dataset, 99.12% on the CT image dataset, which both higher than the other four models. The size of RMT-Net model is only 38.5 M, and the detection speed of X-ray image and CT image is 5.46 ms and 4.12 ms per image, respectively. It is proved that the model can detect and classify COVID-19 with higher accuracy and efficiency.

## Introduction

In recent years, medical images analysis has been widely used in the diagnosis field due to its non-invasive and fast. Traditional manual diagnosis methods are time-consuming and laborious, and each doctor may have different diagnostic principles, resulting in the diversity of diagnosis results. Therefore, automatic classification of coronavirus ID-19 lesions in clinical Settings is quite necessary, which is the motivation of this study.

Recent studies have shown that COVID-19 can be quickly and effectively diagnosed by observing the relevant features of lung CT/X-ray scan images^1–4^. The related algorithms based on deep learning are recognized as the most effective approach to implement image classification of quantitatively and qualitatively with advantages of the workload reduction and misdiagnosis decrease by manual diagnosis^5,6^. On this Background, Many deep learning methods have been used to diagnose COVID-19. The medical image classification method based on CNN has achieved good results. Wang et al.^7^ proposed a lightweight residual projection-expansion-projection extension (PEPX) architecture named COVID-Net. The accuracy of the three classification tasks (COVID-19, normal and pneumonia) is 92.4% , in the four-category task (COVID-19, viral pneumonia, bacterial pneumonia, and normal) is 83.5%. Chen et al.^8^ proposed a lightweight convolutional neural network model named multi-scale gated multi-head attention depth-wise separable CNN(MGMADS-CNN). It achieved accuracy of 96.75% on X-ray images. Song et al.^9^ proposed a Details Relation Extraction neural model (DRE-Net),which is based on the pre-trained ResNet50 and added the Feature Pyramid Network (FPN), to extract the top-K details in the CT images and obtain the image-level predictions. The DRE-Net model performed binary classification experiment (COVID-19 and bacterial pneumonia) on 1485 CT images. The accuracy of model achieved 94.0%. Oulefki et al^10^ proposed a COVID-19 segmentation method, which enhanced image contrast by combining linear and logarithmic splices parameter, and used an image segmentation method to minimize the over-segmentation regions to segment CT tomography images. The method has strong robustness and simplicity with accuracy of 98%. At the same time, Oulefki et al.^11^ proposed a novel 3D visualization segmentation technique based on virtual reality, which has achieved good results in the recognition, measurement and analysis of COVID-19. Pathak et al.^12^ used transfer learning to classify COVID-19. The cost-sensitive top-2 smooth loss function is used to eliminate noise and unbalance of dataset categories. Experimental results show that this method has achieved remarkable classification effect. The above medical image classification method based on CNN mainly uses local spatial information and ignores “global” indication, resulting in sub-optimal performance classification.

In recent years, Vision Transformer has made a breakthrough in the field of computer vision. These models based on global attention have become an effective method of medical diagnosis because they can learn the dependencies of global features. Al et al.^13^ adopted the Vision Transformer architecture as the backbone. The encoder of this algorithm consists of two branches: one to process the original image and the other to process the enhanced original image. Experimental results show that the proposed method is robust in a small amount of training data. Chetoui et al.^14^ fine-tuned several ViT models for multi-class classification problems (COVID-19, pneumonia, and normal cases). Experimental results show that this method is superior to using CNN architecture to detect COVID-19 on CXR images, and can effectively identify infected areas of COVID-19.Yang et al.^15^ proposed covid-vision-transformer (CovidViT), applying transformer architecture and self-focus mechanisms to Covid-19 diagnosis. He used all the output from the encoder to achieve better results, and demonstrated that the transformer-based model was better than CNN at Covid-19 identification. Yaqoob et al.^16^ proposed a deep learning pipeline based on vision transformer that can accurately diagnose COVID-19 from chest CT images. The accuracy rate was 98% on three open source CT scan datasets. Okolo et al.^17^ proposed input enhanced ViT (IEViT). The architecture introduces skip connection, using CNN to output the entire image, and then connecting to the output of each Transformer encoder layer. Experimental results show that the performance of IEViT model is superior to VIT. Cai et al.^18^ proposed Multi-MedVit, a COVID-19 diagnostic framework based on multi-input transformer, and demonstrated that multi-scale data input enhanced data helps improve model stability. Experiments show that the performance of Multi-MedVit is better than that of VGG16, ResNet50 and other CNN-based methods. These literature indicate that transformer has advantages over CNN in the field of medical image classification. However, if only the Transformer structure is used to extract features, the parameters of the network will be greatly increased. In order to combine the advantages of CNN and Vision Transformer, We propose the ResNet Mixed with Transformer (RMT-Net).

RMT-Net integrates Transformer on the basis of ResNet-50 to capture the long-distance dependence relationship in the feature map, and uses convolutional neural network to obtain local features. Depth-wise convolution is introduced in RMT-Net to reduce computation and improve detection speed. The RMT-Net model is only 38.5 M, and the detection speed of X-ray images and CT images is 5.46 ms and 4.12 ms for per image, realizing a high-precision new coronary pneumonia medical image classification algorithm.

It is worth mentioning that our contributions can be summarized as follows

- We propose a CNN-Transformer network structure, which has the ability to capture global features and local features.
- We introduce Depth-wise convolution in the last stage of the network to reduce the number of model parameters.
- We maintain ResNet’s network architecture. The feature extraction capability of the network is improved by reducing the spatial size of features and increasing the number of channels, while the model size is kept within the ideal range.
- We verified the effectiveness of RMT-Net as an image classification algorithm for COVID-19, and achieved good results on both X-ray image datasets and CT image datasets.

The rest of this paper is organized as follows:The “Methodology” Section introduces the details of our proposed RMT-Net, including the overall structure and the mathematical mechanism of each module. In “Dataset preparations” Section, we introduce the experimental environment and datasets. In “Experimental result and analysis” Section, we verify the proposed method and compare it with other models. Finally, we summarize this paper and discuss the possible research direction in the future.

## Methodology

### RMT-Net model

Aiming at the problem of insufficient classification accuracy of COVID-19 X-ray and CT images, this paper proposes a fast and accurate RMT-Net, which is a novel deep learning network based on ResNet-50 merged Transformer.

**Figure 1:**
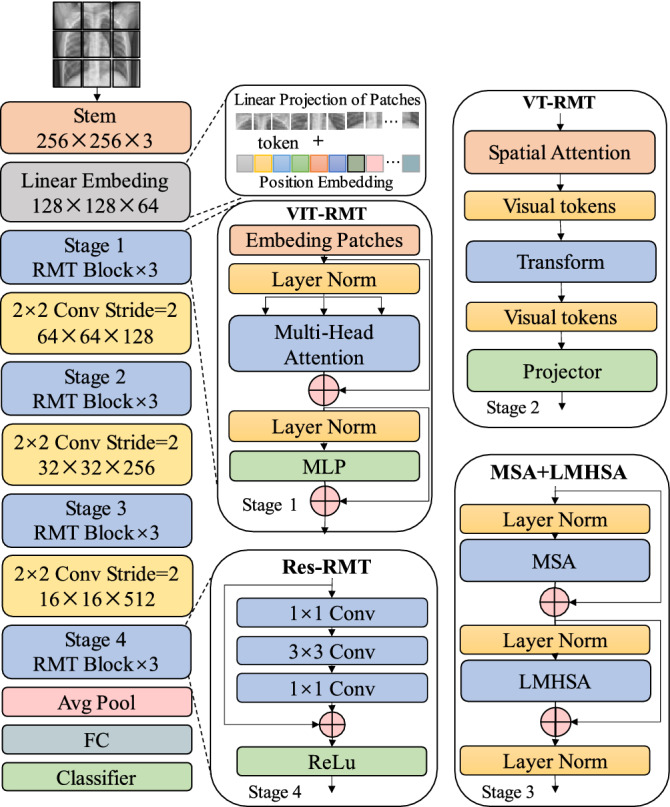
RMT-Net model structure.

The RMT-Net structure is shown in Fig. 1. In order to enhance the migration and generalization ability, RMT-Net adopts the backbone of ResNet-50 with four different stages to extract features with different scales. In order to generate different hierarchical representations in the overall network, we successively stack three stage blocks with the same input resolution to extract features of different scales.

Due to transformer cannot transform the scale of feature map, patch aggregation is adopted to construct downsampling to realize the hierarchical structure of the network. A downsampling is carried out before each stage, which is realized by $2\times 2$ convolution with stride 2. The size of the input image is $256\times 256\times 3$. After the first downsampling of Stem, a $128\times 128$ feature map is obtained, and then a double downsampling operation is performed after each stage. After the average pooling and fully connected layer, the classification results are output.

### Stem

As a basic building block for processing the input data, Stem can preprocess the feature information of the input image, including segmentation, spatial dimension reduction, feature linear transformation and so on. Stem transforms image $x \in R^{H\times W \times C}$into two-dimensional image patches $x_p \in R^{N \times (p^2 \times C)}$ , which can be regarded as $N=(H \times W)\div P^2$flattened two-dimensional sequence blocks, and the dimension of each sequence block is $P^2 \times C$ . Where *P* is the sequence block size and *C* is the feature channel dimension. Position Embedding performs a linear transformation (that is, the fully connected layer) on each two-dimensional sequence, and compresses the two-dimensional sequence into a one-dimensional feature vector.

### Transformer

**Figure 2:**
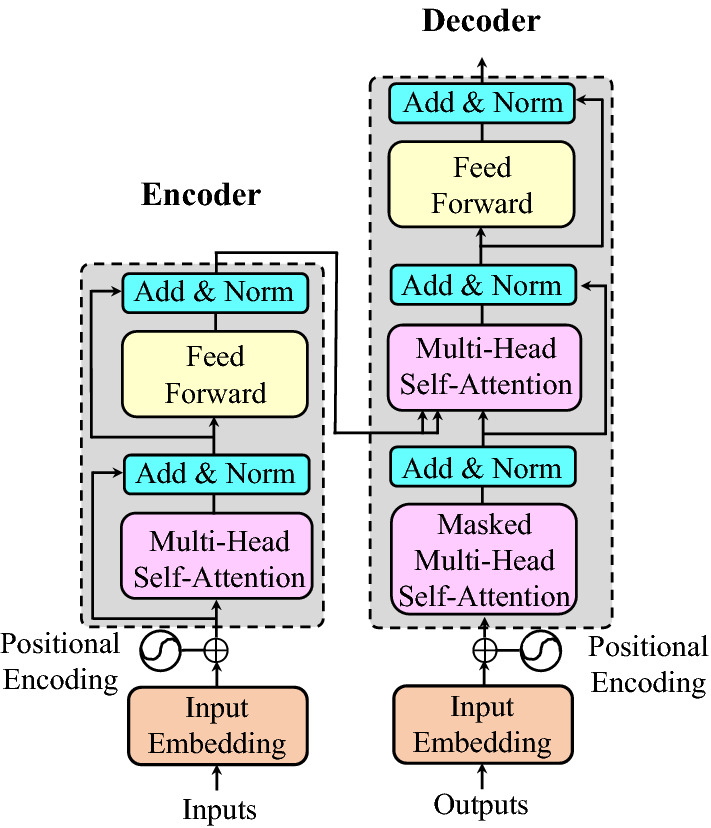
Architecture of the standard Transformer^19^.

Transformer consists of two parts: encoder and decoder. The encoder is mainly composed of multi-head self-attention module and position Feedforward Network (FFN)^19^ . To address the difficulty of training deep networks, Transformer uses a residual connection in each sub-module. For the decoder, the self-attention module in the decoder is adjusted to ensure the order between the output vectors unchanged. The composition of Transformer is shown in Fig. 2. In this paper, Vision Transformer(VIT)^20^ and Visual Transformer(VT)^21^ are mainly used as lightweight Transformer structures, which can reduce the parameters of the model and keep the performance of the model unchanged.

### VIT

VIT is adopted in stage 1 for global feature inference in early stages. In order to obtain a linear input sequence, the input image needs to be divided into patches of fixed size, and linear embedding and position embedding are performed for each patch and then input to the standard Transformer encoder. For image classification, an additional learnable “classification marker” needs to be added to the first position of the sequence before training. The Transformer encoder consists of two modules, Multi-head Self-Attention(MHSA) and Multilayer Perceptron (MLP). Each module adopts residual connection and applies LayerNorm (LN) for normalization. The MLP contains the GELU activation function and two fully connected layers. Equation (1)is the calculation process of each part.

1 $$\begin{aligned} \begin{aligned} Z_0&=[X_{class;X_P^1E;X_P^2E,...;X_P^NE}]+E_{pos} \\ Z_\ell ^{'}&= MSA(LN(Z_{\ell -1}))+Z_{\ell -1} \\ Z_\ell&= MLP(LN(Z_\ell ^{'})) + Z_\ell ^{'} \\ y&= LN(Z_\ell ^{0}) \end{aligned} \end{aligned}$$

where $E\in R^{D\times (P^2\times C)}$ , $E_{pos}\in R^{D\times (N+1)}$. All of the following means that the use of R is a set of real numbers unless otherwise stated. In CNN, each layer feature with locality, two-dimensional neighborhood structure and shift-invariant. In VIT, the self-attention layer is the extracted global features, while only the MLP layer is of local, shift-invariant. Therefore, VIT is used for global feature inference in Stage 1. Compared with CNN, VIT can pay more attention to global features and quickly extract features that are beneficial to the network in the early stage.

### VT

With the deepening of the network, the number of features gradually increases. In order to achieve global feature modeling and reduce network parameters at the same time, VT module is adopted in Stage 2. VT is a new method to represent and process high-level semantics in images. Different from VIT, VT first uses convolutional layer to extract the underlying features. The VT module consists of three steps: (1) Group the features into different semantic concepts to generate a compact set of visual tokens. The grouped semantic information can make the module pay more attention to the semantic information that is beneficial to the network and ignore the useless background information, and then reasonably allocate the computing cost of the entire module. The above operations can be instantiated as Eq. (2).

2 $$\begin{aligned} T=SoftMax_{HW}(XW_A)^TX \end{aligned}$$

where $W_A \in R^{C\times L}$ forms semantic groups from *X*,$SoftMax(\cdot )$ is the softmax activation function,*X*represents the feature map.

For the input feature map *X*, VT uses point convolution to map each pixel $x_p \in R^c$ in the feature map into L groups, and then uses spatial pooling to obtain tokens. All tokens are converted into weights by soxfmax and multiplied with the original feature map *X* to obtain the reassigned attention map. However, many high-level semantic information is sparse in practical applications, and each semantic information may only appear in a few images. Therefore, modeling these high-level semantics independently can be a waste of computational resources. To solve this problem, VT concatenates all layers, so each layer uses the output of the previous layer as input, in this way the visual tokens can be gradually refined. Formally, we can define it as Eq. (3).

3 $$\begin{aligned} \begin{aligned} W_R&= T_{in}W_{T \rightarrow R} \qquad \\ T&= SoftMax_{HW}(XW_R)^T \end{aligned} \end{aligned}$$

Here $$W_{T \rightarrow R} \rightarrow R^{C\times C}$$.

To establish the relationship between semantics, a transformer is applied. It can be expressed by the formula Eq. (4).

4 $$\begin{aligned} \begin{aligned} T_{out}^{'}&= T_{in} + SoftMax_L((T_{in}K)(T_{in}Q)^T)T_{in}\\ T_{out}&= T_{out}^{'} + \sigma (T_{out}^{'}F_1)F_2\qquad \quad \end{aligned} \end{aligned}$$

where$T_{in},T_{out}^{'},T_{out} \in R^{L\times C}$ is Visual Tokens, $(T_{in}K)(T_{inQ})^T \in R^{L\times L}$ is K and Q in Transformers, $F_1,F_2 \in R^{L\times C}$is two point convolution, and $\sigma (\cdot )$ is the relu activation function.

Projecting these visual tokens into the pixel space to obtain the enhanced feature map. As shown in Eq. 5.

5 $$\begin{aligned} X_{out} = X_{in} + SoftMax_L((X_{in}W_Q)(TW_K)^T)T \end{aligned}$$

where $X_{out},X_{in} \in R^{H\times W \times C}$ represents the output and input feature map, $X_{in}W_Q \in R^{H\times W \times C}$ represents the Q value calculated by the input feature map, $(TW_K)\in R^{L\times C}$represents the K value calculated from the token. $W_Q \in R^{C\times C}$ ,$W_K \in R^{C\times C}$ represents the learning weight of Q and K. The result of the multiplication of K and Q determines how the information from visual tokens is projected into the original feature map.

The above is the calculation process of VT. VT can readjust the input feature map according to the semantic importance, and provide the basis for subsequent classification by focusing on favorable semantic information.

### LMHSA

MHSA and Lightweight Multi-head Self-attention (LMHSA) modules are applied in Stage 3 to process the features extracted from the first two stages. The local features are refined by convolution residual blocks to improve the classification accuracy of the network. LMHSA is a lightweight multi-head self-attention model with fewer parameters and easier to deploy than the original MHSA^22^. In order to reduce the amount of computation, LMHSA uses depth-wise convolution with kernel size $k \times k$ and stride *k* to reduce the spatial size of *K* and *V* before performing the attention operation, and uses a learnable relative position bias B when computing MHSA. The calculation process of LMHSA module can be expressed as Eq. 6.

6 $$\begin{aligned} Light \; weight \; Attention (Q,K,V) =SoftMax\left( \frac{QK^T}{\sqrt{d_k}}+B\right) V \end{aligned}$$

where bias $B \in R^{n\times \frac{n}{k^2}}$ is a learnable parameter. The learned relative positional bias can also be transferred to $B^{'} \in R^{m_1 \times m_2}$ of size $m_1 \times m_2$ by bicubic interpolation. MHSA is often applied with multiple LMHSA modules, that is, multiple Lightweight Attention functions (consistent with the number of “heads”) are applied to the input. Each head outputs a sequence of size X, and then concatenates the h sequences into an $n\times d$ sequence, as the output of LMHSA.

## Dataset preparations

### Data collection

**Figure 3:**
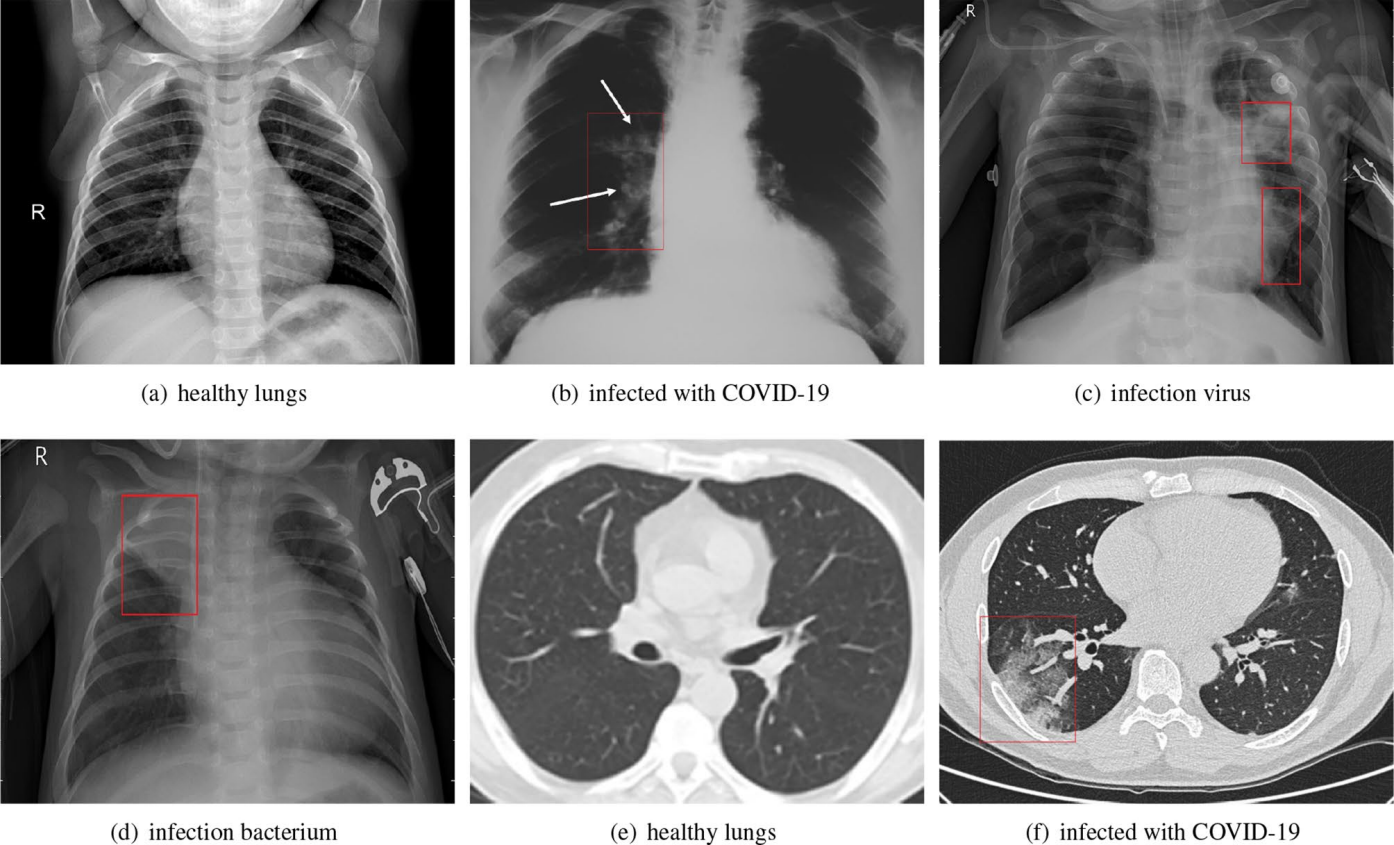
X-ray images (**a**–**d**) and CT images (**e**–**f**).

The datasets used in the experiment were collected from GitHub website^23^, Kaggle website^24^, Kesci website^25^ and Wuhan Tongji Hospital^26^. The above datasets were annotated by hospital experts in a scientific and rigorous manner. The distribution of different samples of COVID-19 X-ray and CT images is shown in Fig. 3. According to the distribution of datasets, X-ray images were classified into four categories: normal, bacterial pneumonia, viral pneumonia and COVID-19 pneumonia, and CT images were classified into two categories: normal and COVID-19 pneumonia. On the basis of previous work^8^ , the dataset is extended with more images. Figure 3a shows the X-ray image of normal lungs, (b) shows the X-ray image of COVID-19 infected lungs, (c) shows the X-ray image of the lung infected with virus, and (d) shows the X-ray image of the lung infected with bacteria. Figure 3e,f show CT sections of normal lung and COVID-19 virus-infected lung.

### Dataset settings

The distribution of collected datasets has the problem of data imbalance, which makes the classifier tend to the class with a large number of samples, which is not conducive to the generalization characteristics and the objective judgment of the model. The data enhancement methods adopted in this paper mainly include affine transformation^27^ , image mirror^28^ and position transformation^29^ . The data distribution before after data enhancement is shown in Table 1.

**Table 1:** The dataset distribution before and after augmentation.

Group	Category	Before	After
Four classes (total: 25,100)	Normal_X	3256	6450
	Virus_ X	3196	6280
	Bacteria _ X	3122	6230
	Covid-19_ X	3089	6140
Binary classes (total: 17,500)	Normal_ CT	4250	8500
	Covid-19_ X	4050	9000

In Table 1, there are 25,100 X-ray images including 6450 normal, 6280 viral pneumonia, 6230 bacterial pneumonia and 6140 with COVID-19 X-ray images. The lung CT images include 8500 normal and 9000 with COVID-19. The augmented dataset can improve the generalization and the reliability abilities of the model. It is significant to enhance the robustness of the model and overcome the imbalance problem of positive and negative samples.

## Experimental result and analysis

The training, validation and testing experiments were undertaken on the platform of Intel Core i7-9700k with Windows 10 64-bit operating system and NVIDIA GeForce GTX 1080Ti GPU. The models are built by deep learning frameworks pytorch 1.9. In order to verify the effectiveness of RMT-Net, another four comparative models (ResNet-50, VGGNet-16, i-CapsNet^30^ and MGMADS-3^8^ are conducted on the declared platform and framework.

### Performance metrics

In this paper, three indicators are used to evaluate the performance of the model. Specificity (TNR)^31^, sensitivity (TPR)^31^ and accuracy (ACC)^31^ can be represented by Eq. (7).

7 $$\begin{aligned} \begin{aligned} TNR&= \frac{TN}{TN+FP}\qquad \\ TPR&= \frac{TP}{TP+FN}\qquad \\ ACC&= \frac{TP+TN}{TP+TN+FP+FN} \end{aligned} \end{aligned}$$

In general, high specificity means a low rate of misdiagnosis, and high sensitivity means a low rate of missed diagnosis. The higher the accuracy, the better the classification effect.

**Figure 4:**
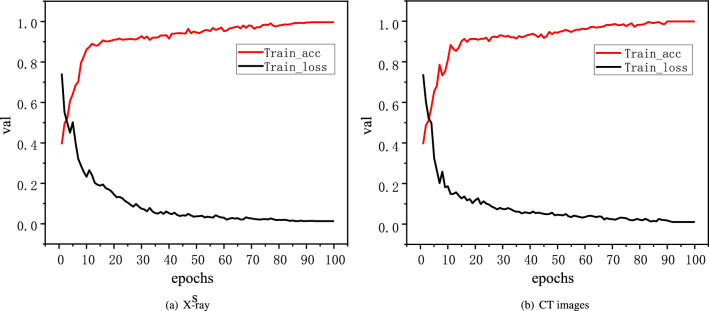
Train_loss and Train_acc curves of X-ray and CT images.

### Training process visualization

In order to visually display the training process of RMT-Net model, the loss values of the first 100 epochs during training on X-ray and CT images were selected for visualization, and the changes of Train_ acc value and Train_ loss value are shown in Fig. 4.

It can be seen that with the progress of training, the Train_ acc and Train_ loss curve drop rapidly, and the RMT-Net can achieve good training results in a short time and basically keep stable. At 100 epoch, the RMT-Net model has a Train_ acc value of 99.64% and a Train_ loss value of 0.0132 on the X-ray image dataset, 99.87% and 0.0102 on the CT image dataset. The RMT-Net model achieves the best training results on both X-ray and CT image datasets. Compared to the other models listed in Table2. The RMT-Net model achieves the best training results on both X-ray and CT image datasets. The trend and amplitude of the curve are excellent, which verifies the stability of the RMT-Net model.

**Table 2:** Comparation to the other four models.

Datasets	Methods	Train_ loss	Train_ acc (%)	Val_ loss	Val_ acc (%)
Four classes (X-ray images)	ResNet-50	0.1987	98.56	0.2018	93.29
	VGGNet-16	0.2145	98.14	0.2453	93.05
	i-CapsNet	0.1584	98.86	0.1862	93.25
	MGMADS-3	0.0139	99.62	0.0140	96.25
	RMT-Net	**0.0132**	**99.64**	**0.0126**	**98.84**
Binary classes (CT images)	ResNet-50	0.1454	99.01	0.1752	96.25
	VGGNet-16	0.1463	98.95	0.1568	93.75
	i-CapsNet	0.1285	98.98	0.1366	95.37
	MGMADS-3	0.0025	99.93	0.0136	98.09
	RMT-Net	0.0102	99.87	**0.0114**	**99.24**

Bold value highlights the gain effect of our method in the table.

It can be seen from Table 2 that in four-classification task of X-ray image, the Val_ loss of RMT-Net is 0.0126, which is lower than the other models. The Val_ acc value of RMT-Net is 98.84%, which is higher than the other models. For binary classification task of CT image , the Val_ loss of RMT-Net is 0.0114 and the Val_ acc is 99.24%. Based on the above content, the RMT-Net model has higher accuracy than the other four models in both training and validation stages, and has a good recognition effect on X-ray and CT images.

### RMT-Net performances tests

In addition to comparing the training and validation results of the model, the evaluation indicators include the model size, specificity, sensitivity and detection accuracy. The comparative experimental results are shown in Table 3.

**Table 3:** The test results of RMT-Net compared to the other four models.

Datasets	Methods	Size(M)	Specificity (%)	Sensitivity (%)	Test _ acc (%)	Speed (ms)
Four classes (X-ray images)	ResNet-50	285	97.24	92.84	93.14	12.24
	VGGNet-16	146	93.54	92.25	92.62	10.09
	i-CapsNet	84	92.62	92.86	93.15	8.58
	MGMADS-3	43.6	98.06	96.60	96.75	6.09
	RMT-Net	**40.8**	**98.26**	**98.08**	**97.65**	**5.46**
Binary classes (CT images)	ResNet-50	275	96.14	95.48	95.25	10.37
	VGGNet-16	154	96.45	94.16	94.38	7.83
	i-CapsNet	82	94.67	95.32	95.62	5.79
	MGMADS-3	43.6	98.17	98.05	98.25	4.23
	RMT-Net	**38.5**	**99.34**	**98.76**	**99.12**	**4.12**

Bold value highlights the gain effect of our method in the table.

As can be seen from Table 3, the model size of RMT-Net is about 40M, which is smaller than the other four models. In terms of model classification performance, the RMT-Net model has higher specificity, sensitivity and accuracy. In X-ray images, the accuracy of RMT-Net on the test set was 96.75%, and its specificity was improved by 1.02%, sensitivity by 5.24%, and accuracy by 4.51% compared with ResNet-50.On CT images, RMT-Net achieved 99.12% accuracy on the test set, with specificity improved by 3.2%, sensitivity improved by 3.28%, and accuracy improved by 3.87% compared to ResNet-50.

### Inference speed

In order to verify whether the reasoning speed of the proposed RMT-Net meets the actual requirement. We conducted a comparison experiment between the proposed RMT-Net and the other four models, and the comparison results are shown in Table 3. The table shows on X-ray image data, the detection speed for each image of ResNet-50, VGGNet-16, i-CapsNet, MGMADS-3 and RMT-Net models is 12.24 ms, 10.09 ms, 8.58 ms, 6.06 ms and 5.46 ms. The detection speed of RMT-Net is clearly faster than the other networks. For example, RMT-Net is 55.4% faster than ResNet-50, 45.9% faster than VGGNet-16, 36.4% faster than i-CapsNet and 9.9% faster than MGMADS-3 . On CT image data, the detection speed are 10.37 ms, 7.83 ms, 5.79 ms, 4.23 ms and 4.12 ms. The detection speed of RMTNet is improved by 60.3% compared with ResNet, 47.4% compared with VGGNet-16, 28.8% compared with i-CapsNet, and 2.6% compared with MGMADS-3.

In addition to the reduction in model size, we believe there are two other factors to improve the speed:(1) The overall structure of RMT-Net is different from that of classic transformer. We adopt pyramid structure, which can greatly increase the computational efficiency of the algorithm by decreasing the spatial dimension step by step. (2) In terms of micro-design, we adopt the lightweight self-attention structure, and adopt the depth-wise convolution in the last stage of the network to further lightweight model. This is one of the reasons for the high computational efficiency of the algorithm.

**Figure 5:**
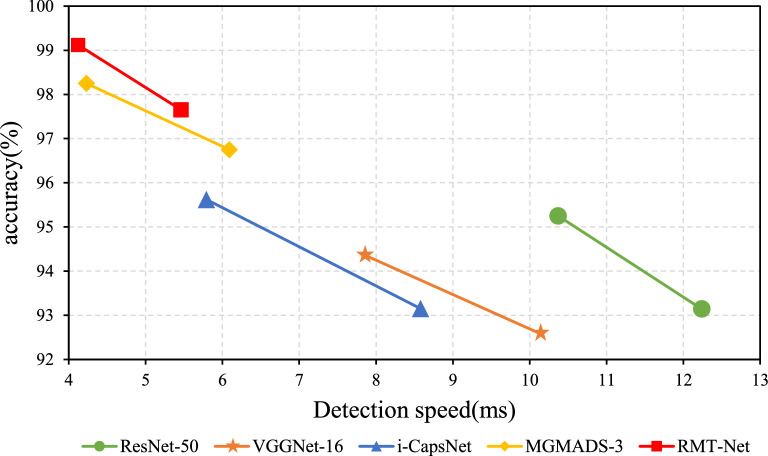
Performance of models on validation set.

Figure 5 shows the speed and accuracy of RMT-Net. It can be seen obviously that the detection speeds are improved to a new level either on X-ray images or CT images. It is further verifed that the proposed model can detect and classify COVID-19 faster.

### Comparison to the related literatures

In order to verify the performance of RMT-Net, this paper compares RMT-Net with other classification models, as shown in Table 4. The numbers in bracket of the third column represents 2, 3, and 4 categories.

As shown in Table 4, the RMT-Net proposed in this paper achieves better classification results than other models in both the four-classification of X-ray images and the second-classification of CT images. In X-ray image classification, the accuracy rate of RMT-Net is 97.65 models.

**Table 4:** Comparisons with related literatures.

Literatures	Methods	Images (classes)	Dataset Quant	Test _acc (%)
Mukherjee^32^	Shallow CNN	X-ray(2)	260	96.92
Abbas^33^	DeTrac	X-ray(2)	1764	95.12
Gupta^34^	COVID-WideNet	X-ray(2)	13,942	91
Hemdan^35^	COVIDX-Net	X-ray(2)	50	91
Apostolopoulos^36^	MobileNet v2(transfer learning)	X-ray(2)	1419	87.02
Wu^37^	ASA-CoroNet	X-ray(3)	994	97.59
Ozturk^38^	DarkCovidNet	X-ray(3)	1442	96.78
Aslan^39^	Deep Learning &Machine Learning	X-ray(3)	2905	96.29
Quan^40^	DenseCapsNet	X-ray(3)	750	90.7
Chen^8^	MGMADS-3	X-ray(4)	17,439	96.75
Wang^7^	COVID-Net	X-ray(4)	13,975	93.3
Khan^41^	CoroNet	X-ray(4)	1300	89.6
Proposed	RMT-Net	X-ray(4)	25,100	97.65
Chen^42^	UNet++	CT(2)	35,355	98.85
Rahimzadeh^43^	Feature Pyramid Network	CT(2)	63,849	98.49
Chen^8^	MGMADS-3	CT(2)	10,839	98.25
Yang^44^	Ednc	CT(2)	2458	97.55
Song^9^	DRE-Net	CT(2)	1485	94.0
Singh^45^	MODE-CNN	CT(2)	150	93.25
Heidarian^46^	COVID-FACT	CT(2)	23,409	90.82
Wang^27^	DeCovNet	CT(2)	630	90.1
Li^47^	COVNet	CT(2)	4356	90
Amyar^48^	Encoder-Decoder with multi-layer perceptron	CT(2)	1044	86
Proposed	RMT-Net	CT(2)	17,500	99.12

### Conclusion

In the paper, a new model named RMT-Net is proposed, which is based on ResNet-50 and Transformer. RMT-Net uses Transformers to capture long-distance dependencies, CNN to obtain local features, and depth-wise convolution to reduce the amount of computation and stage block structure to make the network more scalable, enhance the receptive field and improve the transfer ability. Compared with other classification models, the RMT-Net model shows excellent performance in terms of classification accuracy, model size, and detection speed. With the changes of COVID-19, people are facing great challenges on the unpredictable variations. The X-ray or CT images, or even NMR images can capture more details of the disease, which definitely will enrich the dataset samples, therefore, adaptive network with higher accuracy and faster detection is worthy of further research.

## Data Availability

The datasets generated during and/or analysed during the current study are available from the corresponding author on reasonable request.

## Code availability

*Accession codes.*The proposed RMT-Net backbone network is available publicly for open accessat RMT-Net source.
